# Assessing Hemodynamic Changes During Locoregional Anesthesia in Cesarean Section: The Role of USCOM^®^

**DOI:** 10.3390/diagnostics15222846

**Published:** 2025-11-10

**Authors:** Agnese Lambertini, Sara Doroldi, Stefania Maria Mucci, Silvia Porzio, Fabio Caramelli, Gianluigi Pilu, Elisa Montaguti

**Affiliations:** 1 General and Pediatric Anesthesia and Intensive Care Unit, IRCCS Azienda Ospedaliero-Universitaria di Bologna, 40138 Bologna, Italyfabio.caramelli@aosp.bo.it (F.C.); 2 Obstetric Unit, IRCCS Azienda Ospedaliero-Universitaria di Bologna, Via Massarenti 13, 40138 Bologna, Italy

**Keywords:** USCOM, hemodynamic, cesarean, locoregional anesthesia, pregnancy

## Abstract

**Background**: Locoregional anesthesia (LRA) during cesarean section (CS) is effective but frequently causes hypotension, affecting maternal hemodynamics and fetal outcomes. We investigated whether baseline hemodynamic characteristics predict post-LRA changes, vasopressor needs, and neonatal outcomes. **Methods**: Women undergoing elective CS with LRA were monitored with USCOM^®^ (Ultrasonic Cardiac Output Monitor), recording cardiac output (CO), cardiac index (CI), stroke volume (SV), stroke volume index (SVI), and systemic vascular resistance (SVR) every five minutes. Maternal demographics, vasopressor use, and neonatal outcomes were analyzed using multilevel linear regression. **Results**: LRA caused significant reductions in blood pressure and heart rate (*p* < 0.001). SV initially declined but recovered, while SVR showed minimal variation. Vasopressors were required in 63%, with choice guided by heart rate. Lower baseline SVI predicted greater vasopressor need (37.9 ± 6.7 vs. 34.5 ± 6.6, *p* = 0.050). Lower CO and CI before fetal extraction correlated with reduced neonatal pH, with CI significantly associated with pH < 7.20 (*p* = 0.043). **Conclusions**: USCOM^®^ enables real-time, non-invasive monitoring, supporting individualized management during CS.

## 1. Introduction

Pregnancy induces several essential hemodynamic changes from its early stages, primarily to face the increased demands of utero-placental circulation. These changes include a rise in cardiac output (CO) of up to 50%, driven by increases in both stroke volume (SV) and heart rate (HR), the development of a hypercoagulable state, and a decrease in systemic vascular resistance (SVR) [1,2]. These modifications are crucial for supporting both maternal and fetal well-being throughout pregnancy and during delivery [3], and may be impaired in those pregnancies complicated by hypertensive disorders [4,5].

During cesarean section deliveries, locoregional anesthesia (LRA) is the preferred approach. This typically involves the subarachnoid administration of bupivacaine, sufentanil, and morphine, which provides rapid-onset, stable, and long-lasting analgesia [6]. The analgesic effect of LRA is mediated by blocking unmyelinated C fibers, responsible for transmitting pain signals [7]. However, LRA also has a notable hemodynamic impact. The sympathetic blockade it induces leads to a reduction in SVR and venous return, frequently resulting in hypotension. In response, maternal HR increases to compensate and maintain CO [8]. While the fetus generally has a margin of safety regarding placental perfusion, significant reductions in CO may compromise nutrient supply and lead to fetal acidosis [9]. In such cases, the fetus activates several adaptive mechanisms, including increased oxygen extraction, redistribution of blood flow to vital organs, and a reduction in non-essential activities, to delay the onset of anaerobic metabolism [10].

To manage maternal hypotension during LRA, vasoconstrictive agents such as ephedrine or phenylephrine can be administered, either as boluses or through continuous infusion, alongside volume replacement with crystalloids or colloids [11]. While no single preventive measure—whether volume replacement, vasopressor therapy, or patient positioning—can entirely prevent hypotension, combining these strategies can reduce its severity and mitigate adverse effects [8,12]. Vasopressor administration, in conjunction with goal-directed fluid therapy based on stroke volume, has been shown to alleviate hypotension and improve fetal outcomes [9].

In this context, Ultrasonic Cardiac Output Monitor (USCOM^®^) provides a non-invasive, simple, and reproducible method for assessing maternal hemodynamic parameters [13] during cesarean sections in LRA.

Our study aimed to explore whether anthropometric or baseline hemodynamic characteristics influence hemodynamic changes following LRA induction in elective cesarean sections and if any baseline parameter can anticipate the need for vasopressor therapy and can therefore be used in guiding hypotension control. Furthermore, we investigated the correlation between maternal hemodynamics during surgery and neonatal well-being.

## 2. Materials and Methods

We conducted a prospective monocentric observational study in women who underwent LRA for elective cesarean section. The indications for cesarean section were iterative ones (80%), breech presentation (12%) or maternal choice/tocophobia (8%). We included 60 consecutive singleton pregnancies at term, with an American Society of Anesthesiologists (ASA) score of I-II, and no contraindications to LRA. Exclusion criteria included fetal anomalies or maternal cardiovascular pathology.

In our patients, we recorded hemodynamic parameters using USCOM^®^ at baseline and then every five minutes from LRA induction until the end of the cesarean section, specifically monitoring cardiac output (CO), cardiac index (CI), stroke volume (SV), stroke volume index (SVI) and systemic vascular resistance (SVR). All the hemodynamic assessments were performed by expert trained operators.

USCOM^®^ is a non-invasive Doppler ultrasonic technology for the determination of hemodynamic variables, which combines non-invasiveness and reproducibility. It works through a continuous Doppler transducer that measures the rate of aortic flood flow coming out of the heart. Validated internal algorithms calculate the diameter of the aortic valves based on antropometric parameter of the woman. Then, for the evaluation of the left systolic function, the small transducer is positioned perpendicular to the blood flow out of the heart, at the level of the aortic window on the jugular. The signal recorded is the systolic peak wave coming out of the aorta. From those data, USCOM^®^ calculates how much blood flows per minute, which is the cardiac output, and then the other hemodynamic parameters [14,15].

We collected maternal characteristics (age, body mass index—BMI, parity, and gestational age), details regarding the procedure (maternal heart rate—HR—and both systolic and diastolic blood pressure—SBP and DBP—every 2.5 min, vasopressor therapies administered during the cesarean section, time from LRA induction to fetal extraction), and neonatal outcomes (Apgar scores at 1 and 5 min, umbilical artery and vein pH and base excess, and need for transfer of the neonate to the intensive care unit). The recruitment process did not alter the regular course of care and patients were treated according to standard clinical protocols.

We then compared various subgroups of women according to different baseline anthropometric/hemodynamic variables and analyzed the trend of USCOM^®^ parameters post-LRA, investigating whether different baseline characteristics determined different trends over time.

### 2.1. Ethics

The study was approved by our local ethics committee (015.2019.Oss.AOUBo, 11 October 2018) and a consent form signed at recruitment was obtained from each eligible patient.

### 2.2. Statistics

The mean trajectory of hemodynamic parameters following LRA was assessed using multilevel linear regression analysis with mixed effects [16]. The same model was applied to study the association between baseline characteristics and hemodynamic parameters and their temporal trend during follow-up. All analyses were conducted using Stata 15 software (StataCorp. 2017. Stata Statistical Software: Release 15. College Station, TX: StataCorp LLC, TX, USA). Data analysis was limited to the period before the administration of vasopressor therapies, as their known hemodynamic effects would have influenced subsequent results. Correlations between hemodynamic parameter and neonatal outcomes were analyzed by means of Spearman or Pearson correlations when appropriate.

## 3. Results

The anthropometric characteristics and baseline hemodynamic parameters of the patients are summarized in Table 1.

**Figure 1:**
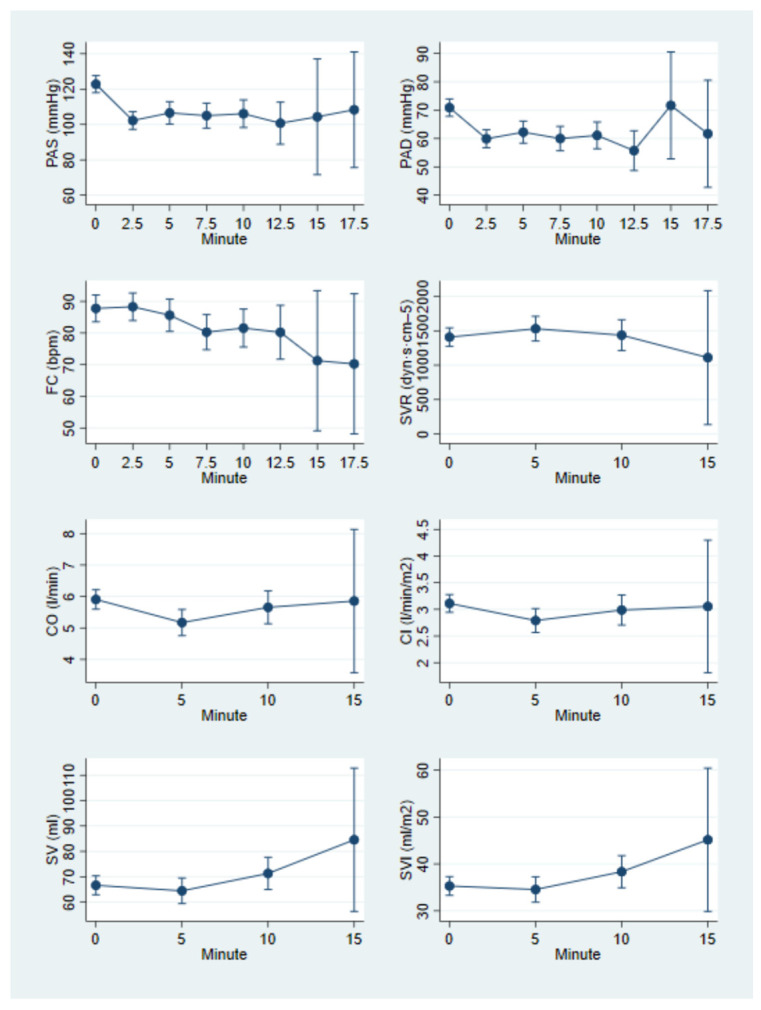
Mean trajectory of hemodynamic parameters following LRA.

After LRA, a significant reduction in both SBP and DBP was observed, along with a decrease in HR (*p* < 0.001). Trends for cardiac output (CO) and cardiac index (CI) showed a tendency to decrease, though this was not statistically significant. Stroke volume (SV) and stroke volume index (SVI) initially decreased at 5 min post-LRA but then showed an increasing trend. Systemic vascular resistance (SVR) demonstrated only minimal variations over time. The mean trajectory of hemodynamic parameters following LRA is shown in Figure 1.

Association between parameters at baseline and during follow-up is detailed in Supplementary Materials.

Age plays an important role in determining a favorable pre-LRA hemodynamic status: women under 35 years of age demonstrated higher CO, CI, and SV values, along with lower SVR and those parameters underwent milder and later changes after LRA procedures. A similar trend is observed for BMI; specifically, a BMI below 30 kg/m^2^ is associated with higher pre-LRA SV, while a body surface area (BSA) below 1.90 m^2^ is linked to higher CI following LRA induction.

**Figure 2:**
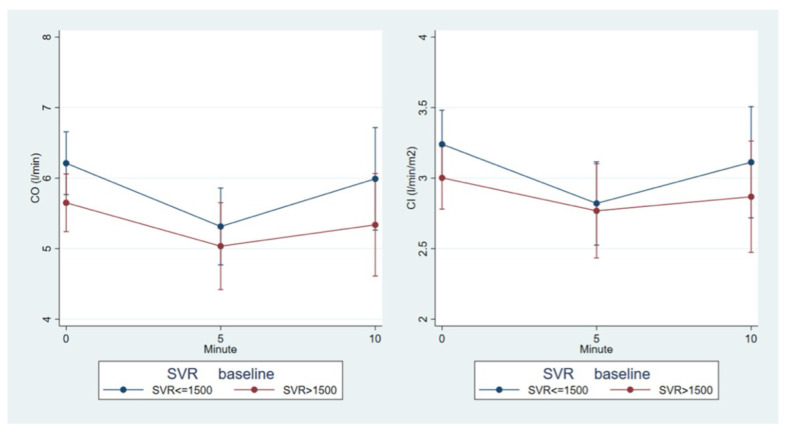
SVR trend according to the baseline CO (<5 L/min, >5 L/min) and baseline CI (<2.8 L/min/m^2^ and >2.8 L/min/m^2^) subgroup.

Unlike other parameters, which follow similar trajectories regardless of baseline values, SVR trends exhibit opposite patterns based on initial SVR and CI values. Specifically, SVR tends to increase in groups with low pre-LRA CI (<2.8 L/min/m^2^) and SVR (<1500 dyn·s·cm^−5)^, while it decreases in groups with higher baseline CI and SVR (Figure 2).

**Figure 3:**
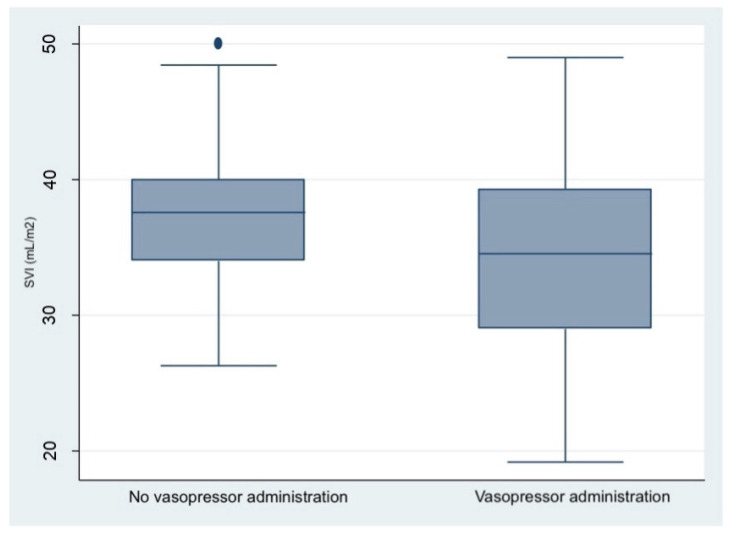
Boxplot showing the distribution of SVI at baseline in the group that received or not any vasopressive therapy.

Vasopressive therapy was administered to 38 patients of our cohort (38/60, 63%), with the choice between ephedrine and phenylephrine depending on HR at the onset of hypotension. Specifically, ephedrine was preferred in instances where HR was <80 bpm due to its vasoconstrictive effect and its ability to increase HR by stimulating both vascular alpha-adrenergic and cardiac beta-adrenergic receptors. Phenylephrine, on the other hand, was chosen for patients with higher HR as it selectively targets alpha-adrenergic receptors without a chronotropic effect. As detailed in Table 2, almost no baseline characteristics were significantly associated with the need for vasopressor therapy. However, women with a lower baseline SVI showed a greater need for support therapies due to hypotension with a borderline statistical significance (*p* = 0.050, Figure 3).

Additionally, we divided the study population into three subgroups based on the vasopressor administered: ephedrine (28 patients), phenylephrine (2 patients), or both (8 patients), to assess the differing impacts of these vasopressors on hemodynamic parameters. Since the group receiving only phenylephrine was very small, we combined it with the ephedrine + phenylephrine group, creating a single subgroup of 10 patients. Changes over time in hemodynamic parameters following the administration of vasopressive therapy are illustrated in Supplementary Materials. In both groups -those treated only with ephedrine (EF) and those treated with phenylephrine with or without ephedrine (FEN)- a significant reduction in SBP and DBP was observed (EF: *p* = 0.001 for SBP and *p* = 0.008 for DBP; FEN: *p* = 0.003 for SBP and *p* = 0.004 for DBP). However, a significant reduction in HR was seen only in the FEN group (*p* < 0.001). No other hemodynamic parameters showed significant modifications after administration of vasopressive therapy.

**Figure 4:**
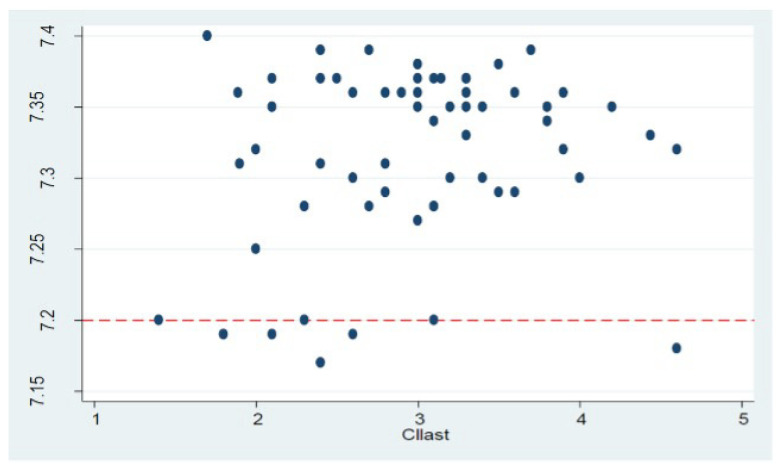
Association between the last maternal cardiac index (CI) measured and neonatal arterial pH. The red line highlights the pH threshold of 7.20.

Regarding neonatal outcomes, we did not register any case of neonatal acidosis nor need for intensive care admission; 8 infants had an arterial pH lower than 7.20. We compared the last values of maternal CO and CI between neonates with a pH > 7.20 and those with pH < 7.20. CO and CI were lower in the group with lower pH, with only CI showing statistical significance (*p* = 0.043). The correlation between the last CI value and neonatal pH is detailed in Figure 4. This relationship was not observed when considering umbilical artery base excess at birth.

## 4. Discussion

In our study, we examined the trends in hemodynamic parameters following LRA during elective cesarean sections and neonatal outcomes. We confirmed the predictable hypotensive effect of LRA on maternal hemodynamics, due to sympathetic block along with vasodilatation effects, with lower impact in younger patients. These findings are already known as predictable effects of subarachnoid anesthesia, which causes an early reduction in blood pressure and CI, due to a decrease of systemic resistance and venous return with a subsequent transitory compensatory tachycardia.

No baseline anthropometric or hemodynamic characteristics assessed by USCOM^®^ device were predictive of the need for vasopressor therapy to manage ALR-induced hypotension, although a lower baseline SVI was associated with a greater need for support therapies. Importantly, in our population, ALR-induced hypotension was not linked to cases of neonatal acidosis; however, lower maternal CI was correlated with neonatal arterial pH values below 7.20.

The use of USCOM^®^ in obstetric practice has largely been limited to the diagnosis and management of hypertensive disorders during pregnancy and preeclampsia [17]. To date, no studies have focused on its application in managing hemodynamic changes following LRA. The effects of LRA on hemodynamic status are well-documented in the literature, and our study confirms these trends: following spinal administration of anesthetics, a sympathetic block is induced, leading to vasodilation (SVR reduction), a reduction in venous return (CO reduction), and the onset of hypotension. Consistent with other studies, we found that these effects are more pronounced in older patients, as the ability to maintain constant SV through HR compensation diminishes with age [8]. Additionally, we observed different SVR trends based on baseline SVR and CI values. Specifically, we suggest that women with higher CI prior to LRA induction—reflecting more intense cardiovascular adrenergic activation—experience a less significant hemodynamic impact following LRA.

As our results show (Figure 1), the most significant drop in blood pressure occurs between 2.5 and 5 min after LRA induction, which coincides with the period during which most vasopressor therapies are required. These findings align with current guidelines, which advocate for early administration of vasopressor therapies to prevent hypotension [18]. We also found that women with lower baseline SVI were more likely to require vasopressor therapies. Since SVI reflects the patient’s volemic status, we speculate that an alternative management strategy could be attempted in these patients. Specifically, in cases of low baseline SVI, fluid resuscitation with crystalloids, rather than vasopressor therapy, may be beneficial [19]. Regarding the hemodynamic differences between ephedrine and phenylephrine, our findings are consistent with previous studies, showing no significant differences in maternal hemodynamics apart from the known reduction in HR following phenylephrine administration [20].

There is some evidence supporting the role of USCOM^®^ in predicting neonatal outcomes. For instance, one study found that in women over 39 years of age, hemodynamic changes such as higher SVR and lower CO were associated with low birth weight [21]. Another study reported significant differences in response to combined anesthesia based on baseline SVR, regardless of blood pressure, emphasizing the important role of SVR in maternal hemodynamics and fetal response. Specifically, fetal heart rate decelerations on cardiotocography were not correlated with maternal blood pressure, while in cases of elevated SVR (>1200 dyn·s·cm^−5^), changes in fetal heart rate were observed, highlighting the importance of SVR in placental perfusion [22]. In a study comparing low versus conventional dose spinal-epidural anesthesia a correlation between maternal CI and neonatal umbilical pH was described, with larger decreases in CI being correlated with lower umbilical venous pH in the conventional dose spinal group. The Authors speculate that there may be a threshold CI below which oxygen transport to the fetus is compromised, leading to the development of acidosis, even if maternal blood pressure remains stable [23]. In our study, neonatal well-being was ensured in all cases of elective cesarean section; the only hemodynamic parameter related to neonatal outcomes was maternal CI: in particular lower maternal CI before extraction was associated with an increased incidence of neonatal arterial pH below 7.20.

The main strength of our study lies in the use of the USCOM^®^ device in an underexplored area of obstetric practice, in a clinical setting where it can be easily integrated into routine monitoring. USCOM^®^ is feasible and non-invasive, making it an ideal tool for tracking hemodynamic changes during cesarean sections without posing additional risk to the mother or fetus. Its ability to provide real-time, continuous data allows clinicians to adjust treatment strategies more effectively during surgery, while reducing patient discomfort compared to more invasive methods.

However, our study has some limitations. The relatively small sample size, especially within certain subgroups, limited the statistical power of our analyses, making it difficult to draw more definitive conclusions. Larger studies are needed to validate our findings and fully explore USCOM^®^’s potential in managing therapies and predicting outcomes during cesarean sections in LRA.

Looking forward, future research could focus on the role of baseline hemodynamic characteristics in guiding treatment decisions, particularly in determining whether volume repletion or vasopressor therapy is the more appropriate intervention in cases of LRA-induced hypotension, especially in patients with low baseline SVI. Given its simplicity and feasibility, USCOM^®^ presents a promising tool for further investigation in both maternal and neonatal care.

## 5. Conclusions

USCOM^®^ is a non-invasive and rapid tool suitable for assessing maternal well-being during cesarean sections under locoregional anesthesia. It may help predict instances of significant hemodynamic compromise and could aid in guiding fluid and drug administration during surgery. Additionally, USCOM^®^ can detect even subtle changes in maternal blood redistribution and compensatory hemodynamic patterns, which also could be useful in predicting neonatal outcomes.

## Figures and Tables

**Table 1:** Baseline anthropometric characteristics and hemodynamic parameters of the cohort (*n* = 60).

Baseline Anthropometric Characteristics and Hemodynamic Parameters	*n* = 60
Age, years	36.2 ± 6.1 [31, 41]
BMI, kg/m^2^	28.0 ± 3.9 [25.7, 29.6]
BSA, m^2^	1.88 ± 0.16 [1.77, 1.97]
Heart rate, bpm	85.9 ± 12.6 [77, 96.5]
Systolic blood pressure, mmHg	127.7 ± 13.2 [120, 137]
Diastolic blood pressure, mmHg	78.3 ± 10.7 [70, 84]
Cardiac output, L/min	5.2 ± 1.3 [4.5, 6.1]
Cardiac index, L/min/m^2^	2.8 ± 0.7 [2.4, 3.1]
Stroke volume, mL	67.4 ± 13.3 [58, 77]
Stroke volume index, mL/m^2^	35.8 ± 6.8 [32, 40]
Systemic vascular resistance, dyn·s·cm^−5^	1595 ± 440.5 [1331, 1807.5]
Arterial pH at birth	7.31 ± 0.06 [7.17, 7.4]
Venous pH at birth	7.28 ± 0.06 [7.02, 7.38]
Arterial BE at birth	−1.7 ± 2.5 [−7.4, 3.0]
Venous BE at birth	−2.0 ± 2.3 [−9.9, 3.0]

Notes: Values are expressed as mean ± standard deviation [interquartile range]. Abbreviations: BMI, Body Mass Index; BSA, Body Surface Area; BE, Base Excess.

**Table 2:** Baseline anthropometric characteristics and hemodynamic parameters in women who did not require vasopressive therapy (*n* = 22) compared to those who required ephedrine and/or phenylephrine (*n* = 38).

Baseline Anthropometric Characteristics and Hemodynamic Parameters	No Vasopressor Therapy *n* = 22	Vasopressor Therapy *n* = 38	*p*-Value
Age, years	36.2 ± 6.4	36.2 ± 6.0	0.992
Weight, kg	73.7 ± 10.6	77.4 ± 11.3	0.208
BMI, kg/m^2^	27.0 ± 3.6	28.6 ± 3.9	0.129
BSA, m^2^	1.88 ± 0.15	1.89 ± 0.16	0.855
Heart rate, bpm	84.6 ± 13.9	86.6 ± 11.9	0.559
Systolic blood pressure, mmHg	129.4 ± 11.7	126.6 ± 14.1	0.443
Diastolic blood pressure, mmHg	80.4 ± 8.9	77.1 ± 11.6	0.243
Cardiac output, L/min	5.3 ± 1.3	5.2 ± 1.3	0.623
Cardiac index, L/min/m^2^	2.8 ± 0.7	2.7 ± 0.7	0.447
Stroke volume, mL	70.1 ± 12.8	65.3 ± 13.3	0.122
Stroke volume index, mL/m^2^	37.9 ± 6.7	34.5 ± 6.6	0.050
Systemic vascular resistance, dyn·s·cm^−5^	1511 ± 310	1643 ± 498	0.212

Notes: Values are expressed as mean ± standard deviation. Abbreviations: BMI, Body Mass Index; BSA, Body Surface Area.

## Data Availability

Data are available upon request due to privacy reason.

## Supplementary Materials

The following supporting information can be downloaded at: https://www.mdpi.com/article/10.3390/diagnostics15222846/s1, Table S1: Variation of Cardiac Output over time and Interaction with anthropometric characteristics and hemodynamic parameters; Table S2: Variation of Cardiac Index over time and Interaction with anthropometric characteristics and hemodynamic parameters; Table S3: Variation of Stroke Volume over time and Interaction with anthropometric characteristics and hemodynamic parameters; Table S4: Variation of Stroke Volume Index over time and Interaction with anthropometric characteristics and hemodynamic parameters; Table S5: Variation of Systemic Vasculare Resistance over time and Interaction with anthropometric characteristics and hemodynamic parameters; Table S6: Comparison of hemodynamic parameters before and after administration of Ephedrine (*n* = 28); Table S7: Comparison of hemodynamic parameters before and after administration of Phenylephrine with or without Ephedrine (*n* = 10).

## Abbreviations

The following abbreviations are used in this manuscript:

CO	Cardiac Output
SV	Stroke Volume
HR	Heart Rate
LRA	Locoregional anesthesia
USCOM	Ultrasonic Cardiac Output Monitor
CI	Cardiac Index
SVI	Stroke Volume Index
SVR	Systemic vascular resistance
