# Supplementary material for: Assessing Hemodynamic Changes During Locoregional Anesthesia in Cesarean Section: The Role of USCOM®
Source: Diagnostics (Basel). 2025 Nov 10;15(22):2846. doi: 10.3390/diagnostics15222846 (PMC12651670; doi:10.3390/diagnostics15222846)
Supplement: Supplementary file 1 [file diagnostics-15-02846-s001.zip › diagnostics-3961938-supplementary.pdf]

Supplementary Table S1. Variation of Cardiac Output over time and Interaction with anthropometric characteristics and hemodynamic parameters

	Mean value at LRA induction	95% CI	Mean value difference<br><br>p-value	Variation per minute after LRA	95% CI	Variation per minute after LRA<br><br>p-value	Difference in variations over time p-value
Overall	5.8	(5.5 – 6.1)	-	-0.04	-0.10 – 0.02	0.157	-
Age ≤35	6.1	(5.7 – 6.6)	0.054	-0.09	(-0.17 – -0.01)	0.035	0.116
Age >35	5.5	(5.1 – 5.9)		0.00	(-0.07 – 0.07)	0.963	
BMI <30	5.8	(5.4 – 6.1)	0.697	-0.03	(-0.09 – 0.03)	0.337	0.479
BMI ≥30	5.9	(5.3 – 6.6)		-0.08	(-0.21 – 0.05)	0.211	
BSA<1.90	5.8	(5.4 – 6.2)	0.971	-0.05	(-0.13 – 0.04)	0.279	0.870
BSA≥1.90	5.8	(5.3 – 6.2)		-0.04	(-0.11 – 0.04)	0.351	
HR ≤85	5.6	(5.2 – 6.0)	0.177	0.00	(-0.07 – 0.07)	0.981	0.089
HR >85	6.0	(5.6 – 6.5)		-0.09	(-0.18 – -0.01)	**0.030**	
SBP ≤130	5.9	(5.6 – 6.3)	0.292	-0.08	(-0.15 – -0.02)	**0.012**	**0.022**
SBP >130	5.6	(5.1 – 6.1)		0.05	(-0.05 – 0.14)	0.325	
DBP ≤80	5.9	(5.5 – 6.2)	0.551	-0.09	(-0.16 – -0.01)	**0.026**	0.095
DBP >80	5.7	(5.2 – 6.2)		0.01	(-0.07 – 0.09)	0.842	
CO <5	5.5	(5.1 – 5.9)	0.072	-0.04	(-0.13 – 0.04)	0.324	0.941
CO ≥5	6.0	(5.6 – 6.4)		-0.04	(-0.11 – 0.03)	0.264	
CI <2.8	5.5	(5.1 – 5.9)	0.075	-0.05	(-0.13 – 0.03)	0.247	0.795
CI ≥2.8	6.0	(5.6 – 6.4)		-0.03	(-0.11 – 0.04)	0.339	
SV <65	5.9	(5.4 – 6.3)	0.618	-0.13	(-0.24 – -0.02)	**0.020**	0.068
SV ≥65	5.7	(5.3 – 6.1)		-0.01	(-0.08 – 0.05)	0.719	
SVI <35	5.9	(5.4 – 6.3)	0.704	-0.08	(-0.19 – 0.02)	0.112	0.321
SVI ≥35	5.7	(5.3 – 6.1)		-0.02	(-0.08 – 0.04)	0.499	
SVR ≤1500	6.0	(5.6 – 6.4)	0.143	-0.04	(-0.11 – 0.04)	0.359	0.842
SVR >1500	5.6	(5.2 – 6.0)		-0.05	(-0.13 – 0.03)	0.252	

Abbreviations: BMI, Body Mass Index; BSA, Body Surface Area; HR, Heart Rate; SBP, Systolic Blood Pressure; DBP, Diastolic Blood Pressure; CO, Cardiac Output; CI, Cardiac Index; SV, Stroke Volume; SVI, Stroke Volume Index; SVR, Systemic Vascular Resistance

Supplementary Table S2. Variation of Cardiac Index over time and Interaction with anthropometric characteristics and hemodynamic parameters

	Mean value at LRA induction	95% CI	Mean value difference<br><br>p-value	Variation per minute after LRA	95% CI	Variation per minute after LRA<br><br>p-value	Difference in variations over time p-value
Overall	3.1	(2.9 – 3.2)	-	-0.02	-0.05 – 0.01	0.221	-
Age ≤35	3.2	(3.0 – 3.4)	0.088	-0.06	(-0.10 – -0.01)	**0.017**	**0.036**
Age >35	2.9	(2.7 – 3.2)		0.01	(-0.03 – 0.05)	0.662	

BMI <30	3.1	(2.9 – 3.3)	0.386	-0.01	(-0.04 – 0.02)	0.524	0.291
BMI ≥30	2.9	(2.6 – 3.3)		-0.05	(-0.12 – 0.02)	0.138	
BSA<1.90	3.2	(3.0 – 3.4)	**0.040**	-0.02	(-0.06 – 0.02)	0.342	0.699
BSA≥1.90	2.9	(2.7 – 3.1)		-0.01	(-0.05 – 0.03)	0.654	
HR ≤85	2.9	(2.7 – 3.1)	0.055	0.01	(-0.03 – 0.05)	0.641	**0.033**
HR >85	3.2	(3.0 – 3.5)		-0.06	(-0.10 – -0.01)	**0.018**	
SBP ≤130	3.1	(2.9 – 3.3)	0.256	-0.05	(-0.08 – -0.01)	**0.015**	**0.016**
SBP >130	2.9	(2.7 – 3.2)		0.03	(-0.02 – 0.08)	0.232	
DBP ≤80	3.1	(2.9 – 3.3)	0.683	-0.04	(-0.08 – 0.00)	0.077	0.230
DBP >80	3.0	(2.7 – 3.3)		0.00	(-0.04 – 0.04)	0.994	
CO <5	2.9	(2.7 – 3.1)	0.129	-0.02	(-0.07 – 0.03)	0.449	0.941
CO ≥5	3.2	(3.0 – 3.4)		-0.02	(-0.06 – 0.02)	0.286	
CI <2.8	2.9	(2.7 – 3.1)	**0.024**	-0.03	(-0.07 – 0.02)	0.217	0.616
CI ≥2.8	3.2	(3.0 – 3.4)		-0.01	(-0.05 – 0.03)	0.501	
SV <65	3.2	(2.9 – 3.4)	0.191	-0.06	(-0.12 – 0.00)	0.048	0.101
SV ≥65	3.0	(2.8– 3.2)		0.00	(-0.04 – 0.03)	0.905	
SVI <35	3.1	(2.8 – 3.3)	0.989	-0.06	(-0.11 – 0.00)	0.044	0.123
SVI ≥35	3.1	(2.9 – 3.3)		-0.00	(-0.04 – 0.03)	0.755	
SVR ≤1500	3.2	(2.9 – 3.4)	0.266	-0.02	(-0.06 – 0.02)	0.357	0.999
SVR >1500	3.0	(2.8 – 3.2)		-0.02	(-0.06 – 0.02)	0.382	

Abbreviations: BMI, Body Mass Index; BSA, Body Surface Area; HR, Heart Rate; SBP, Systolic Blood Pressure; DBP, Diastolic Blood Pressure; CO, Cardiac Output; CI, Cardiac Index; SV, Stroke Volume; SVI, Stroke Volume Index; SVR, Systemic Vascular Resistance

Supplementary Table S3. Variation of Stroke Volume over time and Interaction with anthropometric characteristics and hemodynamic parameters

	Mean value at LRA induction	95% CI	Mean value difference p-value	Variation per minute after LRA	95% CI	Variation per minute after LRA p-value	Difference in variations over time p-value
Overall	65.7	(62 – 69.3)	-	0.4	(-0.3 – 1.1)	0.244	-
Age ≤35	69.4	(64.1 – 74.7)	0.060	0.0	(-1.1 – 1)	0.951	0.255
Age >35	62.5	(57.7 – 67.4)		0.7	(-0.1 – 1.6)	0.093	
BMI <30	64.2	(60.3 – 68.2)	0.130	0.8	(0.1 – 1.5)	**0.031**	**0.020**
BMI ≥30	70.6	(63.3 – 78.4)		-1.2	(-2.7 – 0.3)	0.122	
BSA<1.90	63	(58 – 68)	0.119	-0.7	(-0.3 – 1.7)	0.163	0.374
BSA≥1.90	68.6	(66.5 – 73.8)		-0.1	(-0.8 – 1)	0.845	
HR ≤85	69.5	(64.8 – 74.2)	**0.020**	0.5	(-0.3 – 1.3)	0.239	0.736
HR >85	61.4	(56.4 – 66.4)		0.3	(-0.7 – 1.3)	0.599	
SBP ≤130	67.8	(63.6 – 72.1)	0.095	-0.2	(-1.0 – 0.5)	0.509	**0.007**
SBP >130	61.5	(55.3 – 67.6)		1.6	(0.5 – 2.7)	**0.004**	

DBP ≤80	67.4	(63.0 – 71.7)	0.226	-0.2	(-1.1 – 0.6)	0.586	**0.045**
DBP >80	62.7	(56.5 – 68.9)		1.1	(0.1 – 2.0)	**0.025**	
CO <5	61.8	(56.5 – 67.1)	0.059	0.9	(-0.1 – 1.9)	0.088	0.185
CO ≥5	68.7	(64 – 73.5)		0.0	(-0.8 – 0.8)	0.987	
CI <2.8	63.8	(58.5 – 69.1)	0.361	0.5	(-0.4 – 1.6)	0.272	0.625
CI ≥2.8	67.2	(62.2 – 72.1)		0.2	(-0.6 – 1.1)	0.594	
SV <65	59.8	(54.6 – 65.0)	**0.003**	0.4	(-0.9 – 1.7)	0.553	0.734
SV ≥65	70.2	(65.6 – 74.8)		0.1	(-0.6 – 0.9)	0.739	
SVI <35	63.0	(57.4 – 68.6)	0.229	0.2	(-1.0 – 1.5)	0.680	0.920
SVI ≥35	67.5	(62.8 – 72.1)		0.3	(-0.4 – 1.1)	0.395	
SVR ≤1500	69.0	(63.8 – 74.2)	0.082	-0.2	(-1.1 – 0.6)	0.549	**0.047**
SVR >1500	62.6	(57.7 – 67.5)		1.0	(0.0 – 2.0)	**0.031**	

Abbreviations: BMI, Body Mass Index; BSA, Body Surface Area; HR, Heart Rate; SBP, Systolic Blood Pressure; DBP, Diastolic Blood Pressure; CO, Cardiac Output; CI, Cardiac Index; SV, Stroke Volume; SVI, Stroke Volume Index; SVR, Systemic Vascular Resistance

Supplementary Table S4. Variation of Stroke Volume Index over time and Interaction with anthropometric characteristics and hemodynamic parameters

	Mean value at LRA induction	95% CI	Mean value difference p-value	Variation per minute after LRA	95% CI	Variation per minute after LRA p-value	Difference in variations over time p-value
Overall	34.8	(32.9 – 36.7)	-	0.3	-0.1 – 0.6	0.146	-
Age ≤35	36.7	(33.9 – 39.5)	0.073	-0.1	(-0.6 – 0.5)	0.766	0.095
Age >35	33.2	(30.7 – 35.8)		0.5	(0.1 – 1)	0.026	
BMI <30	34.7	(32.6 – 36.8)	0.811	0.5	(0.1 – 0.9)	**0.012**	**0.012**
BMI ≥30	35.3	(31.3 – 39.2)		-0.7	(-1.5 – 0.2)	0.116	
BSA<1.90	35.4	(32.7 – 38)	0.541	0.5	(-0.1 – 1)	0.092	0.440
BSA≥1.90	34.2	(31.4 – 37)		0.2	(-0.3 – 0.7)	0.507	
HR ≤85	36.5	(34 – 39.0)	0.066	0.3	(-0.1 – 0.8)	0.132	0.522
HR >85	33.0	(30.3 – 35.7)		0.1	(-0.4 – 0.6)	0.686	
SBP ≤130	36.0	(33.7 – 38.2)	0.107	-0.1	(-0.5 – 0.3)	0.709	**0.008**
SBP >130	32.7	(29.4 – 36.0)		0.9	(0.3 – 1.5)	**0.002**	
DBP ≤80	35.5	(33.2 – 37.9)	0.337	0.0	(-0.5 – 0.5)	0.962	0.114
DBP >80	33.5	(30.2 – 36.8)		0.6	(0.0 – 1.1)	0.034	
CO <5	32.7	(30 – 35.5)	0.058	0.6	(0.1 – 1.2)	0.028	0.088
CO ≥5	36.4	(34 – 39)		0.0	(-0.4 – 0.4)	0.993	
CI <2.8	33.1	(30.3 – 36)	0.113	0.3	(-0.1 – 0.9)	0.181	0.559
CI ≥2.8	36.2	(33.6 – 38.8)		0.1	(-0.3 – 0.6)	0.507	
SV <65	32.4	(29.5 – 35.2)	**0.027**	0.3	(-0.3 – 1.0)	0.309	0.549
SV ≥65	36.6	(34.1 – 39.1)		0.1	(-0.3 – 0.5)	0.591	

SVI <35	32.8	(29.8 – 35.7)	0.080	0.1	(-0.5 – 0.9)	0.636	0.862
SVI ≥35	36.2	(33.7 – 38.6)		0.2	(-0.2 – 0.6)	0.283	
SVR ≤1500	36.6	(33.8 – 39.3)	0.073	-0.1	(-0.6 – 0.3)	0.568	**0.024**
SVR >1500	33.1	(30.5 – 35.7)		0.6	(0.1 – 1.1)	**0.010**	

Abbreviations: BMI, Body Mass Index; BSA, Body Surface Area; HR, Heart Rate; SBP, Systolic Blood Pressure; DBP, Diastolic Blood Pressure; CO, Cardiac Output; CI, Cardiac Index; SV, Stroke Volume; SVI, Stroke Volume Index; SVR, Systemic Vascular Resistance

Supplementary Table S5. Variation of Systemic Vasculare Resistance over time and Interaction with anthropometric characteristics and hemodynamic parameters

	Mean value at LRA induction	95% CI	Mean value difference p-value	Variation per minute after LRA	95% CI	Variation per minute after LRA p-value	Difference in variations over time p-value
Overall	1431	(1302 – 1561)	-	4	(-19 – 27)	0.743	-
Age ≤35	1318	(1128 – 1507)	0.115	23	(-12 – 58)	0.199	0.155
Age >35	1524	(1351 – 1698)		-10	(-40 – 19)	0.494	
BMI <30	1427	(1281 – 1573)	0.894	6	(-20 – 31)	0.652	0.716
BMI ≥30	1448	(1170 – 1727)		-5	(-58 – 48)	0.853	
BSA<1.90	1392	(1214 – 1570)	0.525	2	(-32 – 36)	0.929	0.902
BSA≥1.90	1476	(1289 – 1663)		4	(-27 – 36)	0.781	
HR ≤85	1445	(1267 – 1622)	0.840	4	(-25 – 34)	0.772	0.939
HR >85	1418	(1229 – 1607)		3	(-34 – 39)	0.891	
SBP ≤130	1331	(1178 – 1484)	**0.031**	27	(-1 – 54)	0.057	**0.006**
SBP >130	1627	(1406 – 1848)		-38	(-75 – -1)	0.047	
DBP ≤80	1366	(1211 – 1522)	0.202	30	(-1 – 61)	0.058	**0.017**
DBP >80	1542	(1321 – 1763)		-25	(-57 – 8)	0.135	
CO <5	1511	(1322 – 1702)	0.256	11	(-26 – 48)	0.562	0.679
CO ≥5	1363	(1191 – 1535)		1	(-28 – 30)	0.948	
CI <2.8	1541	(1363 – 1718)	0.095	28	(-6 – 62)	0.112	0.089
CI ≥2.8	1332	(1167 – 1499)		-11	(-41 – 18)	0.448	
SV <65	1452	(1257 – 1647)	0.780	6	(-41 – 53)	0.811	0.959
SV ≥65	1415	(1241 – 1588)		4	(-23 – 31)	0.754	
SVI <35	1467	(1265 – 1669)	0.651	4	(-40 – 48)	0.862	0.967
SVI ≥35	1406	(1237 – 1575)		5	(-22 – 32)	0.717	
SVR ≤1500	1216	(1041 – 1391)	**0.001**	9	(-22 – 40)	0.564	0.801
SVR >1500	1621	(1456 – 1786)		3	(-29 – 36)	0.843	

Abbreviations: BMI, Body Mass Index; BSA, Body Surface Area; HR, Heart Rate; SBP, Systolic Blood Pressure; DBP, Diastolic Blood Pressure; CO, Cardiac Output; CI, Cardiac Index; SV, Stroke Volume; SVI, Stroke Volume Index; SVR, Systemic Vascular Resistance

Supplementary Table S6. Comparison of hemodynamic parameters before and after administration of Ephedrine (n=28)

Baseline hemodynamic parameters	Before Administration	After Administration	Difference	p-value
Heart rate, bpm	82 ±21	82 ±20	0 ±20	0.985
Systolic blood pressure, mmHg	90 ± 14	109 ± 25	19 ± 28	0.001
Diastolic blood pressure, mmHg	54 ± 12	64 ± 14	10 ± 18	0.008
Cardiac output, L/min	5.7 ± 1.3	5.6 ± 1.6	-0.1 ± 1.6	0.788
Cardiac index, L/min/m²	2.9 ± 0.7	2.9 ± 0.9	0.0 ± 0.8	0.786
Stroke volume, mL	62 ± 13	67 ± 15	5 ± 19	0.195
Stroke volume index, mL/m²	32.3 ± 7.4	34.9 ± 8.2	2.6 ± 10.9	0.237
Systemic vascular resistance, dyn·s·cm–5	1519 ± 637	1536 ± 529	16 ± 670	0.904

Notes: Values are expressed as mean ±standard deviation

Supplementary Table S7. Comparison of hemodynamic parameters before and after administration of Phenylephrine with or without Ephedrine (n=10)

Baseline hemodynamic parameters	Before Administration	After Administration	Difference	p-value
Heart rate, bpm	103 ± 18	83 ± 17	-20 ± 10	<0.001
Systolic blood pressure, mmHg	86 ± 19	112 ± 22	26 ± 20	0.003
Diastolic blood pressure, mmHg	51 ± 15	67 ± 11	16 ± 13	0.004
Cardiac output, L/min	4.9 ± 1.8	5.2 ± 1.0	0.3 ± 1.9	0.634
Cardiac index, L/min/m²	2.6 ± 1.0	2.8 ± 0.6	0.2 ± 1	0.626
Stroke volume, mL	61 ± 18	65 ± 13	4 ± 21	0.566
Stroke volume index, mL/m²	32.4 ± 9.8	35.0 ± 8.3	2.6 ± 10.8	0.470
Systemic vascular resistance, dyn·s·cm–5	1768 ± 826	1522 ± 386	246 ± 884	0.402

Notes: Values are expressed as mean ± standard deviation
